# Sedentary Lifestyle and Hypertension in a Periurban Area of Mbarara, South Western Uganda: A Population Based Cross Sectional Survey

**DOI:** 10.1155/2018/8253948

**Published:** 2018-05-06

**Authors:** Bruce Twinamasiko, Edward Lukenge, Stella Nabawanga, Winnie Nansalire, Lois Kobusingye, Gad Ruzaaza, Francis Bajunirwe

**Affiliations:** ^1^Faculty of Medicine, Mbarara University of Science and Technology, Mbarara, Uganda; ^2^Department of Community Health, Mbarara University of Science and Technology, Mbarara, Uganda

## Abstract

**Introduction:**

Globally, cardiovascular diseases (CVDs) and diabetes constitute over 50% of the noncommunicable disease (NCD) burden and projections indicate Sub-Saharan Africa will experience a larger burden. Urbanization on the continent is contributing to the change in lifestyle such as diet and physical activity, which may increase the risk for CVDs. There is lack of sufficient data from the African continent on hypertension and its association with sedentary lifestyle.

**Methods:**

We conducted a cross sectional study in periurban Uganda among adults aged at least 35 years. We administered questions on diet, physical activity, and smoking. We took anthropometric measurements, blood pressure, and fasting blood glucose. Hypertension was defined as systolic BP > = 140 and/or diastolic BP > = 90 and/or history of hypertension medications. Logistic regression was used to determine the crude and adjusted odds ratios for the factors associated with hypertension.

**Results:**

We enrolled 310 participants and 50% were female. The prevalence of systolic hypertension was 24.5%, diastolic hypertension was 31%, obesity was 46%, and diabetes was 9%. Of those with hypertension (*n* = 76), 53 participants (69.7%) were not aware they had high BP. Sedentary lifestyle was significantly associated with hypertension even after adjusting for age and obesity.

**Conclusion:**

There is a high prevalence of obesity, hypertension, and diabetes and majority of participants with hypertension are not aware. Participants with a sedentary work style should be targeted for prevention and screening.

## 1. Introduction

The disease burden for noncommunicable diseases (NCDs) is on the rise and now responsible for over 70% of global mortality burden [1]. Cardiovascular disease (CVD) and diabetes are the predominant NCDs and together account for more than half of mortality due to NCDs. The World Health Organization projections indicate NCDs will be responsible for over 80% of global disease burden by 2020 [2] and the largest burden will occur in resource limited settings [3]. Traditionally, infectious diseases have formed the bulk of the disease burden here, but clearly the countries in resource limited settings in Africa are undergoing an epidemiologic transition from a predominantly infectious to noncommunicable disease burden. These countries are now disproportionately affected by the burden of NCDs including CVDs and over 80% of CVD deaths take place in low- and middle-income countries.

The transition may be attributed to changes in lifestyle. Cardiovascular diseases are associated with a sedentary lifestyle [4–6], smoking [7–9], and consumption of a high fat diet [10, 11]. Sub-Saharan Africa is rapidly undergoing urbanization with migration of persons from rural to urban areas [12] and this will likely be accompanied with changes in lifestyle. In addition, the improvements in the economies and standards of living will lead to an increase in life expectancy on the African continent which will also contribute to the increase in the burden of NCDs [13]. Preliminary data from South Africa shows a higher incidence of hypertension and heart disease among black populations in urbanized compared to rural settings [14]. Urbanization in general has been linked to increased risk for NCDs mediated through lifestyle changes [15]. Countries on the African continent need to take strategic action and prepare for this next epidemic appropriately.

A lot of data currently available from the African continent on NCDs are based on projections and few studies have collected empiric data. Secondly, data is required from different settings such as urban, periurban, and rural areas for comparison in order to obtain a more representative picture of disease burden. Many studies have been conducted in rural and urban areas [16–18] but fewer have been conducted in the periurban areas. In Uganda, periurban settlements are densely populated and are occupied by populations that are in transition from rural areas. We hypothesize that these periurban populations may be at an increased risk for changes in lifestyle such as an increased sedentary nature given the migration and hence at risk for NCDs. We also hypothesized that because hypertension is largely asymptomatic, many of the affected persons may be unaware. Given that there is limited research on CVDs in these areas of Sub-Saharan Africa, the aim of this study was to conduct a population based survey in a periurban area of western Uganda to measure the prevalence of hypertension, proportion of those who are aware, and the association between a sedentary lifestyle and hypertension among adults living in this setting. Data from this study will be used to inform programs for prevention of hypertension and cardiovascular disease in general, which are relevant for resource limited settings in Sub-Saharan Africa.

## 2. Methods

### 2.1. Study Design and Setting

We conducted a cross sectional study in Kakoba Division, a periurban location of Mbarara Municipality, south western Uganda. With a population of 40,500 residents, Kakoba Division is the most populous of the six divisions in the Municipality and home to almost 50% of the total municipal population. The area is densely populated and undergoing rapid urbanization; hence the residents here may be representative of those undergoing a transition in the lifestyle. The transition is a change from a predominantly active lifestyle, associated with rural areas to a more sedentary one associated with living in periurban and urban areas in developing countries [19]. Study participants were adults aged 35 years or older and were matched on gender. With about 80% of Uganda's population below 35 years, the country has one of the youngest populations in the world [20].

### 2.2. Sample Size and Sampling

Based on a survey of hypertension in western Uganda [21], we used a proportion of 22%, precision of 5%, and a design effect of 1.2, due to clustering at village level, to calculate a sample size of 310 participants. We obtained the list of parishes and villages from the Kakoba Division offices. Using these lists, multistage sampling was done, first at parish level to randomly select 10 villages. At the selected villages, we obtained the sampling frame of households from the village chairperson, and, from this, we randomly selected 31 households per village to make the total sample size of 310 participants. Research assistants used a guide familiar with the village residents to locate the selected households. At the household level, we assessed members for eligibility and if there was more than one eligible member, lots were drawn to select the study participant. We ensured an equal number of males and females were enrolled, by matching participants by gender.

### 2.3. Inclusion and Exclusion Criteria

Participants were eligible to participate if they were residents of the selected villages for at least 12 months, 35 years or older, able to provide informed consent, and willing to complete study procedures including answering questions on diet, exercise, and medical history, taking an overnight fast and blood glucose test the following day.

### 2.4. Data Collection

Data was collected using a tool adopted from the WHO STEPS questionnaire. We collected demographic and socioeconomic data. We obtained anthropometric measurements with the participants in a standing position, with light clothes on and no shoes. Weight was measured to the nearest 0.1 kg, using a stand on balance, and height to the nearest 0.5 cm, using a stadiometer. Three measurements of blood pressure were taken from the right arm of seated subjects after resting for 5 minutes, using an automatic sphygmomanometer. Fasting blood glucose was measured the following day after the interview. Participants who consented were requested to perform an overnight fast (have dinner before 9 pm and no breakfast) up to the blood draw the following morning. Blood glucose was tested before 10 am using the OneTouch® select glucometer.

### 2.5. Definition of Study Variables

Sedentary work style was defined as having work involving mostly sitting or standing with walking for no more than 10 minutes at a time. Sedentary recreation was defined as recreation or leisure involving mostly sitting, reclining, or standing with no physical activity lasting more than 10 minutes at a time. A standard drink was defined as a bottle of beer, a glass of wine or spirits, a bottle of local brew, a glass of crude liquor, or a pot of local brew. We considered fruit and vegetable intake as not sufficient if consumption was less than five servings per day, as recommended by the World Health Organization [22]. Hypertension was defined as systolic blood pressure ≥ 140 mmHg and/or diastolic blood pressure ≥ 90 mmHg or being on pharmacological treatment for hypertension [23]. Participants were considered to have diabetes mellitus (DM) if they had fasting blood glucose level (FBG) ≥ 7 mmol/L or reported a history of diabetes or use of glucose-lowering drugs [24]. Obesity was defined as having a BMI of ≥ 30 kg/m^2^. We asked about use of tobacco products including cigarettes and smoking a pipe or raw tobacco.

### 2.6. Data Analysis

Statistical analysis was done using STATA version 11 (College Station, Texas). Data were entered into Excel and exported to STATA for analysis. We calculated descriptive statistics using means and standard deviations for uniformly distributed continuous variables such as age, blood pressure, and BMI and nonuniform variables; we calculated medians and interquartile ranges (IQR). We calculated frequencies for categorical variables such as sex and educational level. We use independent samples *t*-test to compare the mean systolic blood pressure between the physical activity categories. We used logistic regression analysis to determine the odds ratios for the factors associated with hypertension and to adjust for confounding factors in the multivariable regression model. We report both the crude (cOR) and adjusted odds (aOR). We aimed to build a parsimonious model with a minimum adjustable set to determine the factors associated with elevated blood pressure. Only variables that were of biological or statistical significance are given emphasis in the discussion of results. We also examined the covariates for collinearity. The level of significance level was set at 0.05. We fit a linear regression model using systolic blood pressure as the outcome to calculate the mean adjusted blood pressure difference between the sedentary and active work categories. We adjusted this model using the set of covariates from the logistic regression model.

### 2.7. Human Subject Issues

We obtained individual informed and written consent from the study participants before study procedures were done. Permission was obtained from the local council village chairperson to conduct sampling and engagement of households. For participants who were not able to read and write, the consent document was read for them and approval to participate obtained using a thumb print. The research protocol was approved by the Mbarara University of Science and Technology Research Ethics committee.

## 3. Results

### 3.1. Description of Study Participants

We enrolled 310 participants, 50% of them were female, and majority were aged 35 to 44 years. The sociodemographic characteristics of the participants are shown in Table 1. Almost 29% (*n* = 88) of the participants had ever smoked and 145 (46.8%) had ever drunk alcohol. The results for the distribution of health behaviour related to cardiovascular disease are shown in Table 2. Among the ever-smokers, the median age at start of smoking was 18 years and IQR of 15–23. Among the current smokers, 9 in 10 of those smoking were smoking daily. Majority reported their work was sedentary (*n* = 191 or 61.6%) and a larger majority reported they spent their leisure time in a sedentary mode (*n* = 213 or 68.7%). Only 36 (11.7%) respondents were aware or had been told by a health worker that they had a high blood pressure.

### 3.2. Frequency of Study Outcomes

The prevalence of systolic hypertension was 24.5%, diastolic hypertension was 31%, obesity was 46%, and diabetes was 9%. Of those with hypertension (*n* = 76), majority (53) of the 76 participants (69.7%) were not aware they had an elevated blood pressure.

### 3.3. Mean Systolic Blood Pressure by Physical Activity Category

The mean systolic blood pressure among those reporting a sedentary work lifestyle was 129.3 compared to 122.6 among those reporting a more active work style. This difference was statistically significant with *p* = 0.0034 and the results are shown in Table 3. The other categories of physical activity were walking or biking for at least 10 minutes continuously to get to and from places and sedentary recreation. None of these showed significant differences in systolic blood pressure.

### 3.4. Factors Associated with Hypertension

We explored several factors to determine those that were significantly associated with elevated blood pressure (hypertension). Age, employment status, smoking status (among ever-smokers), sedentary work style, having been told by health worker that they had an elevated BP in the past 12 months, past diagnosis of diabetes, fasting blood glucose above 6.1, and being obese were all significantly associated with hypertension in the bivariate analysis (Table 4). Older persons were more likely to have a hypertension compared to the younger ones. Compared to respondents in the 35 to 44.9 years' age category, those in the 45 to 55 years' age group had a 2-fold increase in the odds of hypertension and 4.3-fold increase for those above 55 years. Employment was protective of hypertension, with 70% lower odds of hypertension among those employed compared to those not in any form of employment. Among those who had ever smoked (*n* = 90), current smoking was protective of hypertension with 80% lower odds of hypertension compared to the former smokers.

Respondents engaged in work that is predominantly sedentary were 2.7 (95% CI 1.47, 4.88) times more likely to have hypertension compared to those who were not. Similarly, past diagnosis of diabetes, elevated fasting blood glucose, and obesity were all associated with increased odds of hypertension. Several other variables were not significant and these included diet of fruits, vegetables, family history of hypertension, and alcohol consumption.

In the multivariable analysis, our final model comprised age, sedentary work style, and obesity. Older persons and those in a sedentary work style were significantly more likely to have hypertension. Obesity was marginally significant.

### 3.5. Mean Blood Pressure Difference

Participants who had a sedentary work style had higher systolic blood pressure compared to those who were active. The mean adjusted systolic blood pressure difference between the sedentary and active work categories was 6.4 mmHg (95% CI 2.1, 10.7).

## 4. Discussion

The purpose of this study was to determine the association between sedentary lifestyle and hypertension in periurban western Uganda. Sedentary lifestyle, older age, and obesity are associated with hypertension. The data indicate a high prevalence of hypertension, diabetes, obesity, work- and leisure-related sedentary lifestyle, smoking, and reduced fruit and vegetable consumption. Our results are unique because this is one of the few studies to explore factors associated with hypertension among adults from a periurban setting in western Uganda.

Prevalence of diabetes, obesity, and hypertension were all high in this study but not unanticipated. The prevalence of the three conditions is consistent with data from other surveys in Sub-Saharan Africa which increasingly show high prevalence. For hypertension, several studies have been done across the African continent and the prevalence has been quite variable. For instance, the prevalence was below 20% in some surveys [25–27] while some studies showed prevalence about 25%, close to that seen in our study [28–30]. In other studies, prevalence was much higher, above 30% [31]. The findings indicate significant differences in the prevalence due to various reasons and hence there is a need to conduct a meta-analysis to summarize these data. Recently, one has been proposed [32] but has not been conducted as yet. The data will be useful to provide a Sub-Saharan continental summary.

The majority of study participants with hypertension were not aware they had the condition. Almost 70% of the hypertensive patients did not know about their condition. This proportion is extremely high compared to what has been found in other studies such as a recent one from Sri Lanka [33] where the prevalence of undiagnosed hypertension was 31.7%. However, several studies in Sub-Saharan Africa show a very low awareness about hypertension among patients with the condition [34–36]. In fact, a recent meta-analysis of African studies on hypertension showed the pooled prevalence of lack of awareness of hypertension among persons with the condition was 73% [37], consistent with our findings. These staggering statistics of unknown hypertension now call for interventions among these populations.

Our data show the factors significantly associated with hypertension were older age, obesity, and sedentary lifestyle. The findings about these risk factors for hypertension are not surprising and are in agreement with several other studies where age, obesity, and sedentary lifestyle were significant factors for hypertension [28, 33, 38–40]. Of all these factors, obesity and a sedentary lifestyle are modifiable and hence may be used as a basis for interventions to prevent hypertension. In terms of a sedentary lifestyle, our data show that those with an active work style had lower blood pressures compared to those with a sedentary work style. The same observation was not made for recreation or a brief daily physical activity. The data suggest prolonged exposure to physical activity rather than brief sessions in a given day may be more important in lowering blood pressure.

Among ever-smokers, current smoking was associated with lower odds of hypertension compared to those who had quit. This observation is consistent with recent observations that suggest smoking is associated with lower blood pressure [41–43]. This was a subgroup analysis in our study and therefore the sample size was smaller with less power to detect differences. The finding also ceased to be significant after we adjusted for other variables in the multiple regression model. However, this finding requires further investigation in longitudinal studies to establish a potential cause-effect relationship and also explore its mechanism.

Our study has several limitations. First, we conducted a relatively small survey compared to the large national surveys that used similar tools such as ours. However, our sample size was well calculated and suited to answer the primary objective. Second, we did not administer a complete food frequency questionnaire and therefore are unable to comprehensively characterize the diet style for this community and relate the diet style with presence of hypertension. Although our study was based in a rapidly urbanizing periurban area in western Uganda, we were not able to measure the duration of stay in the area to determine how recently migration had occurred. Also, the findings are only generalizable to an African population, given the study setting. Lastly, the cross sectional nature does not allow for establishment of a cause-effect relationship.

Despite the several limitations, our study has an important strength. We conducted this study in a periurban location, an area that clearly is at high risk for noncommunicable diseases due to changes in diet and physical activity. The results from this study are generalizable to several periurban locations in Sub-Saharan African countries that are undergoing rapid urbanization.

## 5. Conclusion

In conclusion, our study has shown that, in this periurban area of western Uganda, there is a high prevalence of obesity, hypertension, and diabetes. Majority of participants with hypertension are not aware. Participants with a sedentary work style are more likely to have hypertension compared to the active ones. We recommend that routine measuring of blood pressure should be done to detect hypertension, more vegetables and fruits should be included in the diet, and a more active work and leisure lifestyle might help to protect from hypertension. Larger population based longitudinal studies in rapidly urbanizing areas of Sub-Saharan Africa should be conducted to understand risk factors in these largely unstudied populations.

## Figures and Tables

**Table 1:** Sociodemographic characteristics of study participants in Kakoba Division of Mbarara Municipality (*n* = 310).

Characteristic	*n* (%)
Sex	
Male	155 (50.0)
Female	155 (50.0)
Age categories (years)	
35–44.9	178 (57.4)
45–54.9	87 (28.1)
>55	45 (14.5)
Marital status	
Married monogamously	199 (64.1)
Married polygamous	38 (12.3)
Divorced/separated	25 (8.1)
Widow/widower	30 (9.7)
Never married	18 (5.8)
Level of education	
No formal schooling	39 (12.6)
Primary school level	154 (49.7)
O Level	73 (23.5)
A level	30 (9.7)
Tertiary	14 (4.5)
Occupation	
Peasant farming	54 (17.4)
Business/trading	190 (61.3)
Professional	14 (4.5)
Manual/casual labor	52 (16.8)

**Table 2:** Frequency of health behaviours related to cardiovascular disease in Kakoba Division, Mbarara Municipality.

Characteristics	*n* (%)
Ever smoked tobacco products	
Yes	90 (28.4)
No	220 (71.6)
Current smoking status (among ever smoked, *n* = 90)	
Still smoking	25 (27.8)
Stopped smoking	65 (72.2)
Ever consumed alcoholic drink	
Yes	145 (46.8)
No	165 (53.2)
Alcohol in the past 12 months (among ever drank, *n* = 143)	
Yes	83 (58.1)
No	60 (41.9)
Frequency of having at least one standard drink in the last 12 months (*n* = 83, among alcohol drinkers in 12 months)	
5 or more days per week	26 (31.3)
1–4 days per week	31 (37.4)
1–3 days/month	26 (31.3)
Number of days that fruits are eaten per week, median (IQR)	2 (1–3)
Number of servings of fruits, mean (SD)	1.04 (0.48)
Number of days vegetables are eaten per week	
None	45 (14.5)
1-2	143 (46.1)
3–7	122 (39.4)
Number of servings of vegetables, mean (SD)	1.1 (0.5)
Sedentary work style	
Yes	191 (61.6)
No	119 (38.4)
Sedentary recreation/leisure	
Yes	213 (68.7)
No	97 (31.3)
Told by health worker that you have high blood pressure in the past 12 months	
Yes	36 (11.7)
No	272 (88.3)
Had cholesterol level measured in the last 12 months	
Yes	1 (0.3)
No	309 (99.7)
Told by health worker that had a high cholesterol level in the past 12 months	
Yes	1 (0.3)
No	309 (99.7)

**Table 3:** Comparison of mean systolic blood pressure by physical activity status among residents of Kakoba, western Uganda.

Category of physical activity	Mean systolic BP	95% CI	*p* value
Sedentary work style			
No	122.6	119.3, 125.9	**0.0034**
Yes	129.3	126.4, 132.1	
Sedentary recreation			
No	128.4	123.6, 132.8	0.37
Yes	126.1	123.6, 128.5	
Walks or pedals bicycle at least 10 minutes continuously daily			
No	128.2	124.7, 131.6	0.28
Yes	125.8	122.8, 128.6	
Vigorous work among those *not* sedentary at work (*n* = 112)			
Not vigorous work	120.9	115.9, 125.9	0.29
Vigorous work	124.7	119.7, 129.7	

In-bold results = significant at 0.05 level.

**Table 4:** Bivariate and multivariate analysis of factors associated with hypertension.

Variable	cOR (95% CI), *p* value	aOR (95% CI), *p* value
Age (years) category		
35–44.9	1	1
45–54.9	**1.98 (1.08**–**3.65)**, **0.027**	**2.1 (1.14, 3.98)**, **0.018**
>55	**4.31 (2.13**–**8.74)**, **<0.001**	**4.5 (2.18, 9.44)**, **<0.001**
Sex		
Female	1	- - -
Male	1.33 (0.79, 2.26), 0.27	
Employment status		
Not working	1	- - -
Self employed	**0.32 (0.15**–**0.69)**, **0.003**	
Employed	**0.30 (0.10**–**0.90)**, **0.032**	
Ever smoked		
No	1	- - -
Yes	0.24 (0.06, 1.1), 0.063	
Current smoking status (among ever smoked, *n* = 90)		
Stopped smoking	1	- - -
Currently smoking	**0.18 (0.39**–**0.85)**, **0.03**	
Sedentary work style		
No	1	1
Yes	**2.68 (1.47**–**4.88)**, **0.001**	**2.7 (1.48, 5.12)**, **0.001**
Told you have elevated BP by health worker in the last 12 months		
No	1	- - -
Yes	**7.31 (3.48**–**15.37)**, **0.001**	
Past diagnosis of diabetes		
No	1	- - -
Yes	**3.79 (1.32**–**10.88)**, **0.013**	
Seen a traditional healer for diabetes in the past 12 months		
No	1	- - -
Yes	**15.0 (1.03**–**218.3)**, **0.047**	
Fasting blood glucose		
<6.1 mmol/l	1	- - -
6.1–6.9 mmol/l	**4.55 (1.99**–**10.43)**, **<0.001**	
>7.0 mmol/l	2.05 (0.90–4.68), 0.087	
Obesity (BMI >= 30)		
No	1	1
Yes	**1.75 (1.04**–**2.94)**, **0.036**	1.72 (0.99, 2.99), 0.052

In-bold results = significant at 0.05 level.

## Abbreviations

CVDs: Cardiovascular diseases
NCDs: Noncommunicable diseases
WHO: World Health Organization
BP: Blood pressure
FBG: Fasting blood glucose
BMI: Body mass index.
